# Modeling porosity loss in Fe^0^-based permeable reactive barriers with Faraday’s law

**DOI:** 10.1038/s41598-021-96599-8

**Published:** 2021-08-20

**Authors:** Huichen Yang, Rui Hu, Hans Ruppert, Chicgoua Noubactep

**Affiliations:** 1 Angewandte Geologie, University of Göttingen, Goldschmidtstraße 3, 37077 Göttingen, Germany; 2 School of Earth Science and Engineering, Hohai University, Fo Cheng Xi Road 8, Nanjing, 211100 People’s Republic of China; 3 Department of Sedimentology and Environmental Geology, University of Göttingen, Goldschmidtstraße 3, 37077 Göttingen, Germany; 4 Centre for Modern Indian Studies (CeMIS), University of Göttingen, Waldweg 26, 37073 Göttingen, Germany

**Keywords:** Environmental sciences, Hydrology

## Abstract

Solid iron corrosion products (FeCPs), continuously generated from iron corrosion in Fe^0^-based permeable reactive barriers (PRB) at pH > 4.5, can lead to significant porosity loss and possibility of system’s failure. To avoid such failure and to estimate the long-term performance of PRBs, reliable models are required. In this study, a mathematical model is presented to describe the porosity change of a hypothetical Fe^0^-based PRB through-flowed by deionized water. The porosity loss is solely caused by iron corrosion process. The new model is based on Faraday’s Law and considers the iron surface passivation. Experimental results from literature were used to calibrate the parameters of the model. The derived iron corrosion rates (2.60 mmol/(kg day), 2.07 mmol/(kg day) and 1.77 mmol/(kg day)) are significantly larger than the corrosion rate used in previous modeling studies (0.4 mmol/(kg day)). This suggests that the previous models have underestimated the impact of in-situ generated FeCPs on the porosity loss. The model results show that the assumptions for the iron corrosion rates on basis of a first-order dependency on iron surface area are only valid when no iron surface passivation is considered. The simulations demonstrate that volume-expansion by Fe^0^ corrosion products alone can cause a great extent of porosity loss and suggests careful evaluation of the iron corrosion process in individual Fe^0^-based PRB.

## Introduction

Permeable reactive barriers (PRBs) are an in-situ technology for remediation of contaminated groundwater^1–4^. It consists of subsurface filters filled with reactive materials to clean through-flowing polluted groundwater. PRB containing granular metallic iron (Fe^0^) has been demonstrated to be a promising, economically-feasible and environmentally-friendly technology for groundwater remediation^5–9^. Polluted water with a broad range of chemical species such as halogenated organics^10^, nitroaromatics^11,12^, dyes^13^, phenolic compounds^14^, heavy metals^15^ and various oxyanions^16,17^ can be efficiently treated by applying metallic iron-based permeable reactive barrier.

Although the performance of Fe^0^-based PRBs are generally satisfactory, questions remain on the long-term effectiveness of PRBs, which are expected to operate for decades^18–20^. Permeability loss is one key of concern. Researchers have reported that the main cause of permeability loss is the reduction in pore space caused by mineral precipitation on the surface of Fe^0^^21–29^. Clogging of the pore space in the reactive zone reduces the porosity and hydraulic conductivity of the reactive medium, which can result in preferential flow patterns, bypassing and changes in residence time^20,30^.

At pH 4.5–8.5, which is the typical rage of PRBs operation, there is continuing aqueous iron corrosion at the surface of Fe^0^. The chemical composition of the FeCPs depends upon the local pH–Eh conditions under which the reaction takes place^31^. All the possible corrosion products have much less density compared to the parent metal, which makes the iron corrosion a highly volumetric expansive process^32,33^. Depending on the level of oxidation, iron may expand by as much as six times its original volume^34^. Therefore, the very first cause of permeability loss in Fe^0^-based PRBs is pore filling with iron corrosion products^35^.

The expansive nature of iron corrosion has been properly considered in reinforced concrete (RC) industry. In that context, the volumetric expansion of the iron induces internal pressure on the surrounding concrete, causing the cracking of cover concrete and affecting the service life of the structures^36^. A number of investigations have been conducted for the study of the cracking of cover concrete induced by corrosion^37–39^. On the contrary, in the Fe^0^-PRB literature, iron volumetric expansion process has not been properly considered^35^. A phenomenological model was established by Kouznetsova et al.^40^ to estimate the long-term performance of Fe^0^. The model described the decline of iron reactivity as a function of space and time by observing the degradation of chlorinated ethanes, but did not consider iron corrosion processes. Numerous detailed geochemical models were proposed to simulate the chemical reactions and flow transport inside Fe^0^-PRB and the effect of mineral precipitation on hydraulic properties of PRBs^20,30,41–43^. The iron corrosion rate in these models is expressed with a first-order dependence on iron surface area, and the rate coefficient was derived from the report of Reardon^44^. According to the modeling results, it is perceived that the permeability loss is mainly caused by foreign precipitates (e.g. CaCO_3_) or mixed precipitates (e.g. with FeCO_3_)^20^. Moreover, iron surface passivation is not considered in previous studies. However, above pH ~ 4.8 and in an oxygen containing aqueous environment the generated porous oxide layers contains three-valent iron causing strong inhibition effect on the iron corrosion^45^. In this study, Iron surface passivation is described as linear or parabolic growth of corrosion products^46,47^.

The aging behavior due to corrosion of iron particles was investigated most of the time using column tests or by measuring the hydrogen pressure build-up in long-term batch studies^44,48–50^. According to previous studies, the estimated life-time of iron granular particles ranged from several years to several decades^51,52^. Some studies assumed that iron particles will be completely consumed in the reaction of groundwater and estimated the life-time of Fe^0^-based PRB by the iron mass and iron corrosion rate^53–56^. This approach to estimate the PRB service life is only valid, if the initial PRB pore volume and the used Fe^0^/aggregate ratio enable complete Fe^0^ depletion^35,57^. In this study, the residual amount of Fe^0^ and the residual pore space of the PRB are calculated on a time-line to evaluate this life-time estimation method.

Therefore, in this study, a mathematical model is formulated to study the porosity loss of Fe^0^-based PRB solely caused by the volumetric expansive corrosion of iron based on Faraday’s Law including iron surface passivation. For simplification, iron corrosion in the deionized (DI) water is considered. Based on the results of Luo et al.^58^, which show the porosity change of iron exposed to deionized water, our model is calibrated in order to simulate the porosity loss for long-term operation.

## Fundamental of Fe^0^/H_2_O system

Since Fe^0^ is not stable under environmental conditions, and the redox couple H^+^/H_2_(E_0_ = 0.00 V) is higher than that of Fe^II^/Fe^0^(E_0_ = − 0.44 V) at a_H+_ = 1^59,60^, a transfer of electrons from the Fe^0^ body (solid state) to the Fe/H_2_O interface occurs whenever a Fe^0^ specimen is immersed in an aqueous solution^59,61,62^. Equations (1a) and (1b) show that the oxidative dissolution of Fe^0^ by protons (H^+^) from water (H_2_O $\Leftrightarrow$ H^+^ + OH^−^) forms Fe^2+^ and Fe(OH)_2_ by increasing the pH. In the presence of dissolved oxygen, Fe^2+^ and Fe(OH)_2_ can be oxidized to less soluble Fe(OH)_3_ (Eqs. 2a, 2b). Fe(OH)_2_ and Fe(OH)_3_ are polymerized and further transformed to various oxyhydroxides (Eq. 3)^59,63–65^. Equation (4) summarizes the process of aqueous iron corrosion.

1a $${\text{Fe}}^{0} + 2{\text{H}}^{ + } \Rightarrow {\text{Fe}}^{{2 + }} + {\text{H}}_{2}$$

1b $${\text{Fe}}^{0} + {\text{2H}}_{{2}} {\text{O}}^{ - } \Rightarrow {\text{Fe}}\left( {{\text{OH}}} \right)_{{2}} + {\text{H}}_{{2}}$$

2a $${\text{4Fe}}^{{{2} + }} + {\text{O}}_{{2}} + {1}0{\text{H}}_{{2}} {\text{O}} \Rightarrow {\text{4Fe}}\left( {{\text{OH}}} \right)_{{3}} + {\text{8H}}^{ + }$$

2b $${\text{4Fe}}\left( {{\text{OH}}} \right)_{{2}} + {\text{O}}_{{2}} + {\text{2H}}_{{2}} {\text{O}} \Rightarrow {\text{4Fe}}\left( {{\text{OH}}} \right)_{{3}}$$

3 $${\text{Fe}}\left( {{\text{OH}}} \right)_{{2}} ,{\text{Fe}}\left( {{\text{OH}}} \right)_{{3}} \Rightarrow {\text{FeO}},{\text{Fe}}_{{3}} {\text{O}}_{{4}} ,{\text{Fe}}_{{2}} {\text{O}}_{{3}} ,{\text{FeOOH}},{\text{Fe}}\left( {{\text{OH}}} \right)_{{3}}$$

4

**Figure 1:**
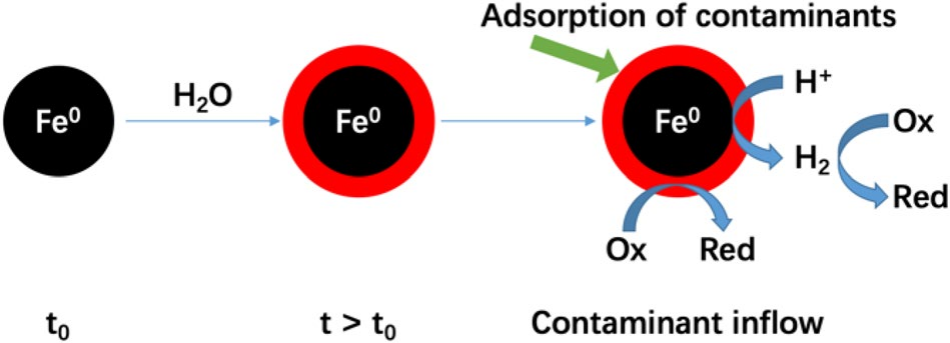
Principle of contaminant removal process in Fe^0^/H_2_O system.

Comprehensive research on Fe^0^ for water treatment revealed that the generation of iron oxyhydroxides (iron corrosion products or FeCPs) is the basis of contaminant removal in Fe^0^/H_2_O systems^7,8,60,66^. Figure 1 depicts the principle of contaminant removal process in Fe^0^/H_2_O system. The electrochemical corrosion of immersed Fe^0^ induces the generation of reducing agents, i.e. Fe^2+^, Fe(OH)_2_ and H_2_ (Eqs. 1a, 1b). The generated iron oxyhydroxides on Fe^0^ (red layer) is an adsorbent for contaminants^64^, as well as a contaminant scavenger (Eqs. 2a, 2b).

However, the generation of FeCPs has the side effect of being expansive. Thus, modeling the volume-expansion process is essential to design an efficient and sustainable Fe^0^/H_2_O filter system.

## Modeling porosity loss in Fe^0^-based PRB

### Description of the model based on Faraday’s Law

Faraday’s Law describes the fundamental quantitative relationship of redox partners in electrochemistry. It depicts the relationship between the amount of material reacting during electrochemical reactions according to the average current and the total reaction time^67^. Equation (5) summarizes Faraday’s Law:

5 $$m=\frac{QM}{Fz},$$

where $m$ is the mass of the substance liberated or deposited at the electrode (g); $Q$ is the total electric charge (Coulombs or Amperes seconds)); $M$ is the molar mass of the substance (g/mol)$, F$ is the Faraday constant; and $z$ is the valency of an ion formed from the reacting substance.

For Fe^0^ oxidative dissolution electrochemical reaction (Eqs. 1a, 1b), Eq. (5) can be transformed by using Eqs. (6) and (7) into Eq. (8):

6 $$Q=It,$$

7 $$I=iA,$$

8 $$\partial {V}_{iron}=\frac{M}{zF\rho }A\cdot i\cdot \partial t,$$

where $I$ is the current (Ampere), $t$ the reaction time (s), $i$ the current density (Ampere/m^2^) and $A$ the surface area of iron (m^2^). ${\partial V}_{iron}$ is the volume depletion of iron, $\rho$ the density of iron = 7.85 × 10^3^ kg/m^3^, $M$ = 55.85 g/mol, $F$ = 96,500 C/mol; $z$ is taken equal to 2 (Eq. 1a).

Assuming the iron particle is a sphere, we obtain for one iron particle

9 $$\partial {V}_{iron}\approx A\cdot \partial {r}_{iron},$$

where $\partial {r}_{iron}$ is the radius depletion of the iron particle. Combining Eq. (8) and Eq. (9), we get:

10 $$\frac{\partial {r}_{iron}}{\partial t}=\frac{M}{zF\rho }\cdot i,$$

where $\frac{\partial {r}_{iron}}{\partial t}$ is the corrosion rate (in mm/year) and $\frac{M}{zF\rho }$ is a constant. So the corrosion rate (in mm/year) and the current density are linearly related.

### Calculation of the coefficient of volumetric expansion

As discussed above, the generation of FeCPs is a volumetric-expansion process. A coefficient of volumetric expansion ($\upeta$) is introduced to describe this behavior. Equation (11) states the definition of $\upeta$:

11 $${V}_{oxide}=\eta {V}_{iron},$$

where ${\text{V}}_{\text{oxide}}$ is the volume of the generated FeCPs. The change of volume and radius can be described as follows:

12 $${\partial V}_{expansion}={\partial V}_{oxide}-\partial {V}_{iron}=\left(\eta -1\right){\partial V}_{iron},$$

13 $${\partial r}_{expansion}={\partial r}_{oxide}-\partial {r}_{iron}=\left(\eta -1\right){\partial r}_{iron},$$

where ${\text{V}}_{\text{expansion}}$ is the expansion volume, ${r}_{oxide}$ is the increased radius with the generation of FeCPs and ${\text{r}}_{\text{expansion}}$ is the expansion radius of the iron particle.

If we combine Eq. (8) and Eq. (12), we have the expression of the total volume change ($\Delta V$) over time as:

14 $$\Delta V={V}_{expansion}={\int }_{0}^{t}\left(\eta -1\right)\frac{M}{zF\rho }\cdot A\cdot i\cdot \partial t.$$

Due to the complexity of the iron corrosion product, $\eta$ varies with different corrosion environment and Fe^0^ intrinsic reactivity^65^. Table 1 depicts the volumetric expansion coefficients of different possible corrosion products based on the study of Caré et al.^33^.

**Table 1:** Volumetric expansion coefficients of possible corrosion products.

Phase	Name	Volumetric expansion coefficient ($\upeta$)
Fe(OH)_3_	Fe^III^ hydroxide	4.53
FeCO_3_	Siderite	3.86
Fe(OH)_2_	Fe^II^ hydroxide	3.47
${\upalpha}$-FeOOH	Goethite	2.67
${\upalpha}$-Fe_2_O_3_	Hematite	1.98
$\upgamma$-Fe_2_O_3_	Maghemite	1.91
Fe_3_O_4_	Magnetite	1.97

### Estimate of growth of corrosion products and passivation

The growth of corrosion products may follow a linear or parabolic law depending on the metal properties and geochemical conditions^46,47^. Due the complexity of iron corrosion, both linear and parabolic law are considered in this study.

For a metal that the generated oxide film is not protective, which means that no passivation occurs, the rate of growth of oxide film remains constant:

15 $$y=kt,$$

where $k$ is a constant, $y$ is the film thickness (m) and $t$ is the corrosion time.

For a metal that forms a protective oxide film,

16 $${y}^{2}=kt.$$

However, the relationship between corrosion rate and time is not so simple. The following equation is usually used^68^:

17 $${y}^{n}=kt,$$

where n is the coefficient of passivation. The value of n is usually larger than 1. In case of iron corrosion in air or corrosion in soil, n ranges from 1 to 3 depending on the suppression of diffusion of oxygen through the formed oxide film^68^.

The coefficients of passivation n are taken equal to 1, 1.5 and 2 respectively in this study. For n = 1, the rate of growth of oxide film on the iron surface remains constant and the passivation of Fe^0^ is not considered. For n = 2, it means that iron passivation occurs during the corrosion process and the generated corrosion products form a protective oxide film. For the case n = 1.5, iron passivation occurs with time but the oxide film on the surface of iron is not completely protective.

Combining Eq. (10) and Eq. (13), we have:

18 $${\text{y}} = \int {{\text{r}}_{{{\text{oxide}}}} } = \int {\frac{{{\text{M}}\upeta }}{{{\text{zF}}\uprho }} \cdot {\text{i}} \cdot \partial {\text{t}}} .$$

For n = 1, with Eqs. (15) and (18), we obtain:

19 $${\text{y}} = \int {\frac{{{\text{M}}\upeta }}{{{\text{zF}}\uprho }} \cdot {\text{i}} \cdot \partial {\text{t}}} = {\text{kt}}$$

From the right part of Eq. (19) we can conclude, that when no passivation is considered, the current density ($\text{i}$) and corrosion rate (in mm/year) (Eq. 10) are constants.

If we consider iron passivation occurs in the system, Eqs. (17) and (18) transform into:

20 $${\text{y}}^{{\text{n}}} = \left( {\int {\frac{{{\text{M}}\upeta }}{{{\text{zF}}\uprho }} \cdot {\text{i}} \cdot \partial {\text{t}}} } \right)^{{\text{n}}} = {\text{kt}}.$$

For n = 2, from Eq. (20), we get:

21 $${\text{i}} = \frac{{{\text{k}}^{{0.5}} }}{{\frac{{{\text{M}}\upeta }}{{{\text{zF}}\uprho }}}} \cdot {\text{t}}^{{ - 0.5}} = \alpha \cdot {\text{t}}^{{ - 0.5}} ,$$

where ${\upalpha}$ is a constant. For n = 1.5, Eq. (20) simplifies into:

22 $$\text{i}=\upbeta \cdot {\text{t}}^{-\frac{1}{3}},$$

where $\upbeta$ is a constant.

### Estimate of surface area and porosity change

The iron surface area is constantly changing during the operation. The area changes can be calculated by the following equations:

23 $$A=N\cdot 4\pi {r}^{2}=N\cdot 4\pi {\left({r}_{0}-{r}_{depletion}\right)}^{2},$$

24 $${r}_{depletion}={\int }_{0}^{t}\frac{M}{zF\rho }\cdot i\cdot \partial t,$$

where $\text{N}$ is the total iron particle number, ${\text{r}}_{0}$ is the initial radium of iron particles.

The total iron particle number can be calculated by Eq. (25):

25 $$N = \frac{{V_{solid}^{0} \cdot \tau_{iron} }}{{{\raise0.7ex\hbox{$4$} \!\mathord{\left/ {\vphantom {4 3}}\right.\kern-\nulldelimiterspace} \!\lower0.7ex\hbox{$3$}} \cdot \pi r_{0}^{3} }},$$

where ${\text{V}}_{\text{solid}}^{0}$ is the initial volume occupied by the solid particles (i.e. iron and sand particles), and ${\uptau }_{\text{iron}}$ is the initial iron volume ratio.

The porosity of the system ($\Phi$) can be described as follow:

26 $$\Phi =\frac{1-{V}_{solid}}{{V}_{reactive\;zone}}=1-\frac{{V}_{solid}^{0}+\Delta V}{{V}_{reactive\;zone}},$$

where ${\text{V}}_{\text{reactive zone}}$ is the volume of the reactive zone, and $\Delta \text{i}$ is the total volume change (Eq. 14).

### Model assumptions

**Figure 2:**
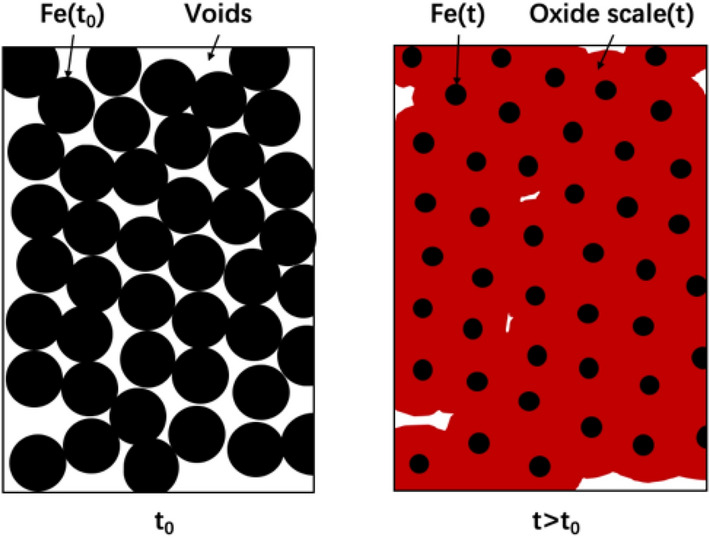
Illustration of the reactive zone before (left) and after corrosion of a layer of metallic iron (right).

A simplified illustration of the model is shown in Fig. 2. As iron constantly transforms in the water, the radius of the iron particle decreases with time. In the meanwhile, the generated FeCPs, which have larger volumes than the Fe^0^, fill the pore space and cause the porosity loss in the system. The following assumptions are made in this model.

- All iron particles are spheres and have identical radium, which is taken equal to 1 mm.
- Uniform Fe^0^ corrosion: the radius reduction of spherical Fe^0^ particles is the same for all particles.
- The volume of the reactive zone remains constant.
- Fe^0^ corrosion products progressively fill the available pore space.

### Model calibration

The results of Luo et al.^58^ are used to calibrate the current density in this model. The corrosion rate (in mm/year) can be obtained with the calibrated current density value (Eq. 10).

In Luo et al.’s study, iron/sand mixtures were packed between two layers of sand in the column experiments. Three iron mixing ratios (100%, 50%, 10%, w/w) of the barrier material were tested for different water type (a synthetic groundwater, acidic drainage and deionized (DI) water). The porosity data for only 100% Fe^0^ columns which tested with DI water were reported. Since this study considers the condition that iron corrodes in DI water, the porosity change data for 100% Fe^0^ column reacted with DI water were used to calibrate the parameters of the model. These parameters were then used to simulate the porosity loss in the systems with different iron mixing ratios.

The authors reported a porosity loss from 57 to 32% when 100% Fe^0^ column are exposed to deionized water within 16 days under 2 mL/min flow velocity. The flow condition is 56 times greater than the average flow rate at a typical PRB installation^69^. In order to estimate the real operation of PRBs, the aging of the fast flow experiment have to be scaled to real time conditions. The surface-loading rate was used in the calculation. At this fast-flow rate, 16 days represents an equivalent reaction period of 2.2 years (800 days) under typical field conditions, as the surface-loading rate is proportional to flow velocity^48^. This prediction does not include the effect of kinetics on precipitation due to increased velocity and reduced residence time, and the operating life of PRBs will be overestimated using results from fast flow rate experiment^48,49^.

The grain size was reported as between 2.38 and 0.30 mm. Since a uniform particle radius is assumed in this study, the grain size taken was equal to 2 mm.

### Model results

#### Corrosion rates for different coefficients of passivation

**Figure 3:**
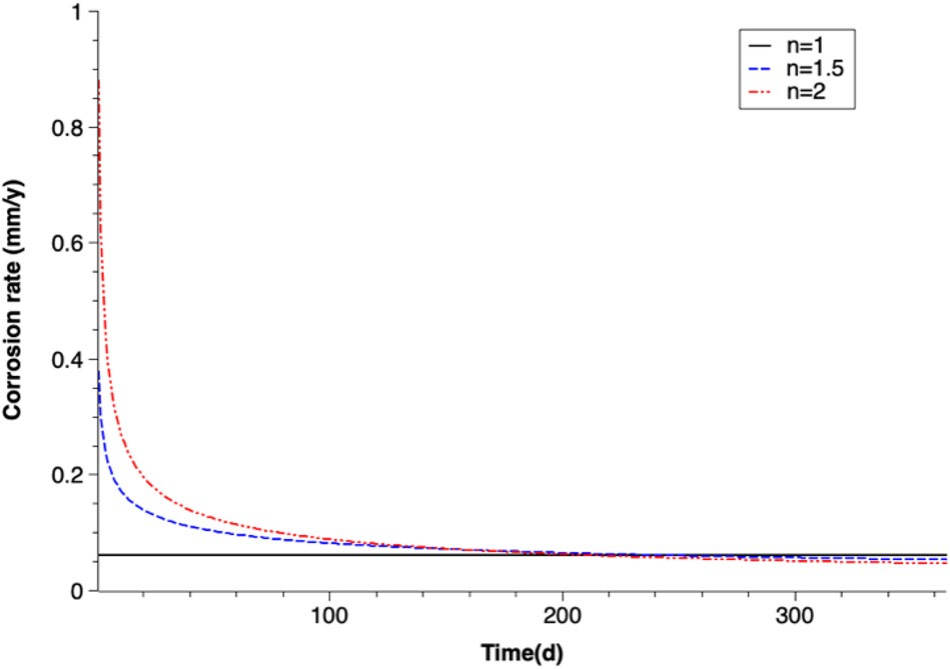
Corrosion rates versus time (n is the coefficient of passivation) assuming goethite is the corrosion product.

The current density (i) is the calibrated parameter in this study. The corrosion rate value can be then calculated (Eq. 10). The corrosion rate (in mm/year) is either a constant value or a function of the corrosion time when different coefficients of passivation (n) are taken. The derived corrosion rate values are shown in Fig. 3. It is assumed that goethite (FeOOH) is the only corrosion product, which is reported by Luo et al.^58^ on the basis of SEM images and EDX spectra results.

The corrosion rate (in mm/year) is a constant (= 0.06 mm/year) if the passivation of iron corrosion is neglected. When the passivation of iron is considered ($\text{n}>1$), dramatic variations of the calibrated corrosion rate values are detected in the beginning phase of corrosion. The initial corrosion rate values are significantly large, which are 0.88 mm/year for the coefficient of passivation n = 2 and 0.38 mm/year for n = 1.5. The rates decrease rapidly and reach a relatively stable value after 200 days of corrosion. The average stable corrosion rate value is 0.055 mm/year.

#### Relative porosity loss for different coefficients of passivation

**Figure 4:**
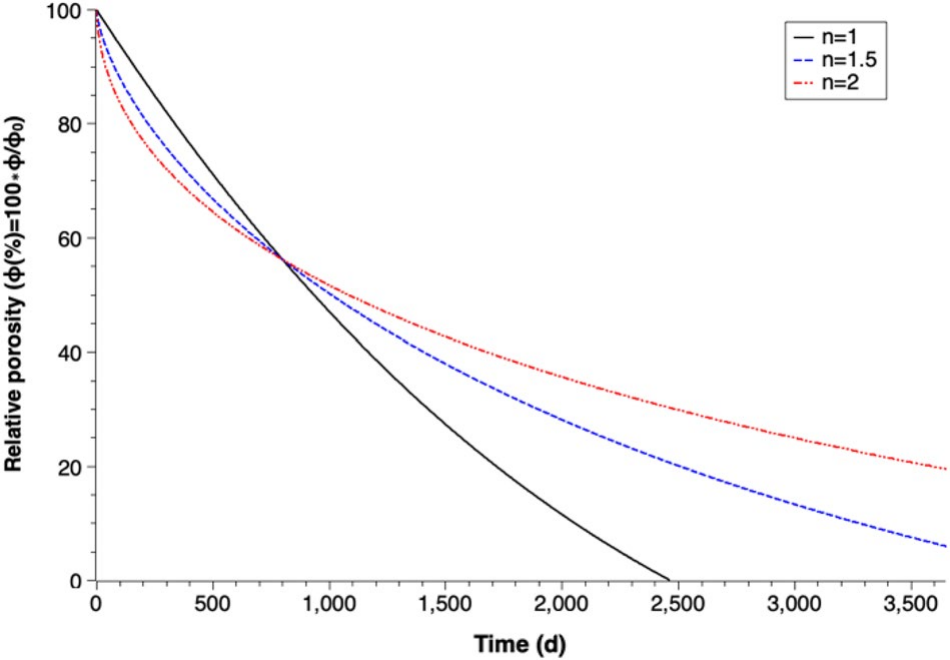
Percentage decrease of relative porosity through the formation of goethite over time for different coefficients of passivation (n).

Figure 4 depicts the simulations of long-term porosity changes for different corrosion patterns. It is assumed that goethite is the only corrosion product. The Y-axis in the figure represents the relative porosity of the system, which can be described as

27 $$\text{Relative\;porosity}=\frac{\Phi }{{\Phi }_{0}}$$

where $\Phi$ is the porosity at time t and ${\Phi }_{0}$ is the initial porosity of the system.

Significant porosity losses can be detected in all three simulations (Fig. 4). The simulation with constant corrosion rate (in mm/year) shows the most remarkable porosity reduction, which decreases to 0 on day 2464. Zero relative porosity means there is no pore space left in the barrier, i.e. no underground water can flow through the PRB. Thus, the PRB has no water remediation effect after day 2464.

The relative porosity values of three simulations show dissimilar features along corrosion pathway and also divergent results after long-term simulation. After 10 years of simulation, the relative porosity values decrease to 5.94% for coefficient of passivation of n = 1.5 and 19.50% for n = 2. The simulation with higher coefficient of passivation (n) shows a more rapid porosity loss in the beginning phase but only slight porosity change after long-term corrosion. This indicates that the rate of diffusion process decreases with the increase of the thickness of the generated corrosion products. The differences among the three simulation results indicate that the iron passivation is an important factor determining porosity for Fe^0^-based PRBs’ long-term performance estimation.

#### Relative porosity loss for different iron mixing ratios

**Figure 5:**
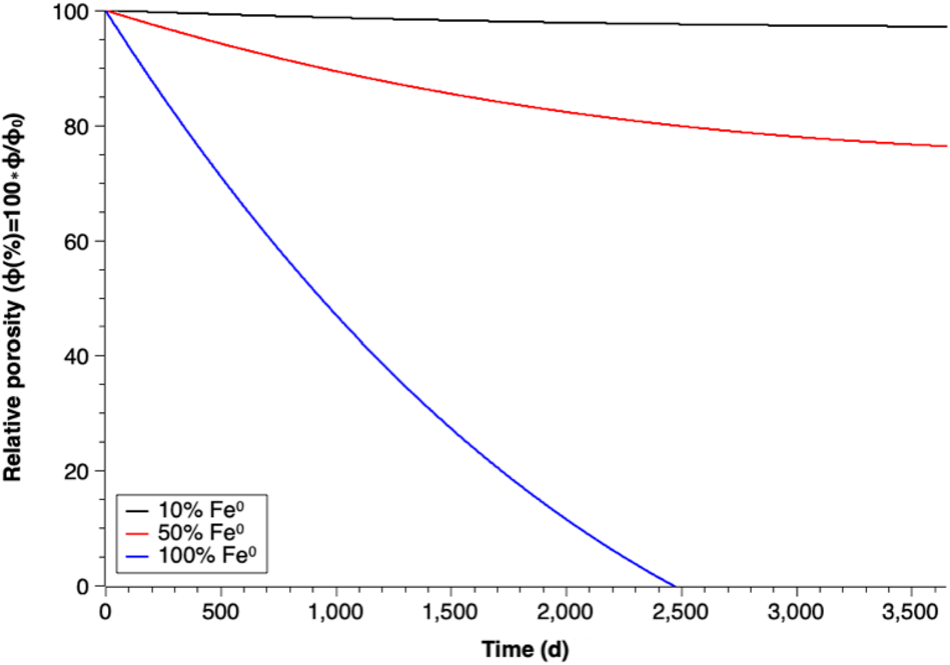
Percentage decrease of relative porosity through the formation of goethite over time for different iron mixing ratios and n = 1.

The calculated corrosion rates were utilized to simulate the porosity loss in the systems with different iron mixing ratios. Figure 5 depicts the relative porosity change along time with iron mixing ratios of 10%, 50% and 100% (W/W). It can be seen that a lower percentage of Fe^0^ within the barrier shows less porosity reduction during long-term operation.

#### Relative porosity loss for different corrosion products

**Figure 6:**
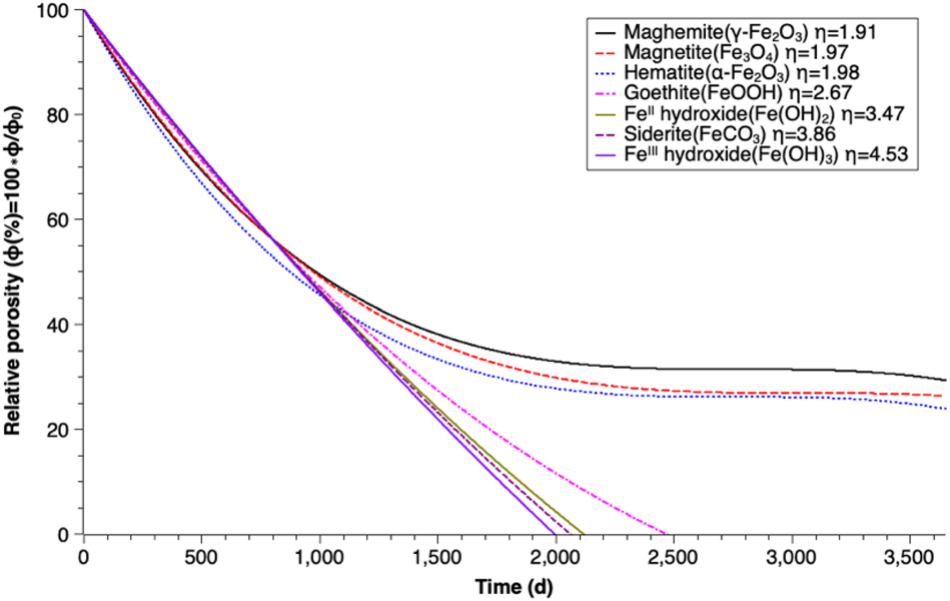
Percentage decrease of relative porosity over time for different corrosion products ($\upeta$ is the coefficient of volumetric expansion) for n = 1.

The previous simulations in this study all assume that goethite is the only corrosion product of iron corrosion. However, in the real corrosion process, other FeCPs may form. Figure 6 illustrates the porosity loss simulations for different possible corrosion products with no iron passivation considered. All possible iron corrosion products have a larger volume than the corroded iron. In general, the simulations with higher coefficients of volumetric expansion ($\upeta$) show stronger porosity reduction. The results of the simulations imply that iron corrosion products have an important effect on the porosity reduction of the PRB system.

## Implications for the estimation of the durability of Fe^0^-based PRB

**Figure 7:**
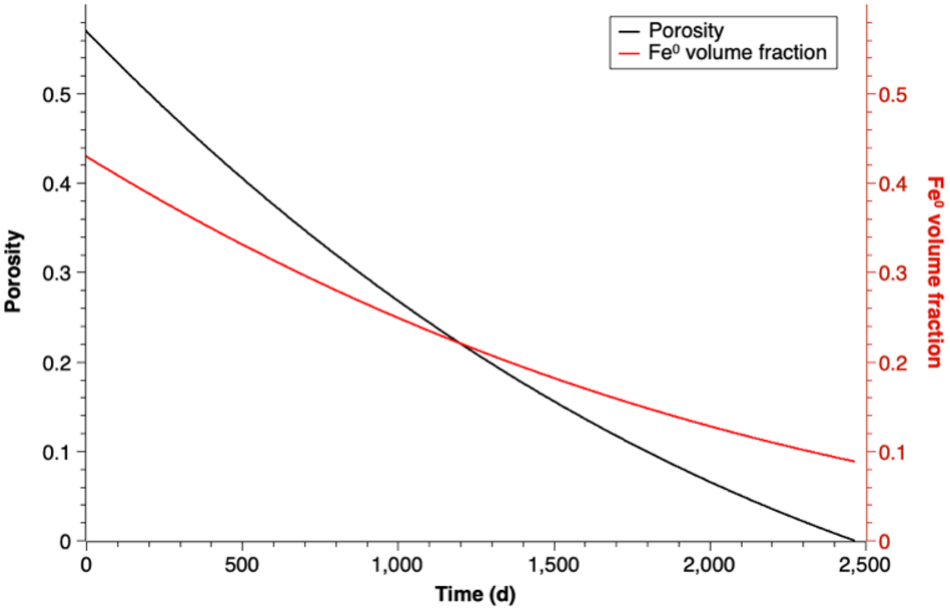
Porosity and Fe^0^ volume fraction versus time assuming goethite as reaction product.

Figure 7 shows the calculated porosity and Fe^0^ volume decrease with time by the formation of goethite. According to simulation results, when the porosity value of the simulation with constant corrosion rate (in mm/year) reaches 0, there is still 0.09 m^3^/m^3^ Fe^0^ volume fraction left in the system. It means the PRB system loses its capability to remove contaminants before the iron is completely consumed. Therefore, the previous method to estimate the lifetime of Fe^0^ on basis of the corrosion rate^56^, which assumes the iron will be totally oxidized, cannot be used to estimate the service lifetime of iron-based PRB systems. Moreover, this study simulates only the contact of Fe^0^ and deionized water and considers merely the effect of expansive volume of iron corrosion. If the geochemical condition changes, e.g. the solution has high calcium concentration, which will cause additional mineral precipitation in the iron zone and trigger even larger porosity loss, an earlier failure of PRB technique can be expected.

## Comparison of corrosion rates in different studies

**Figure 8:**
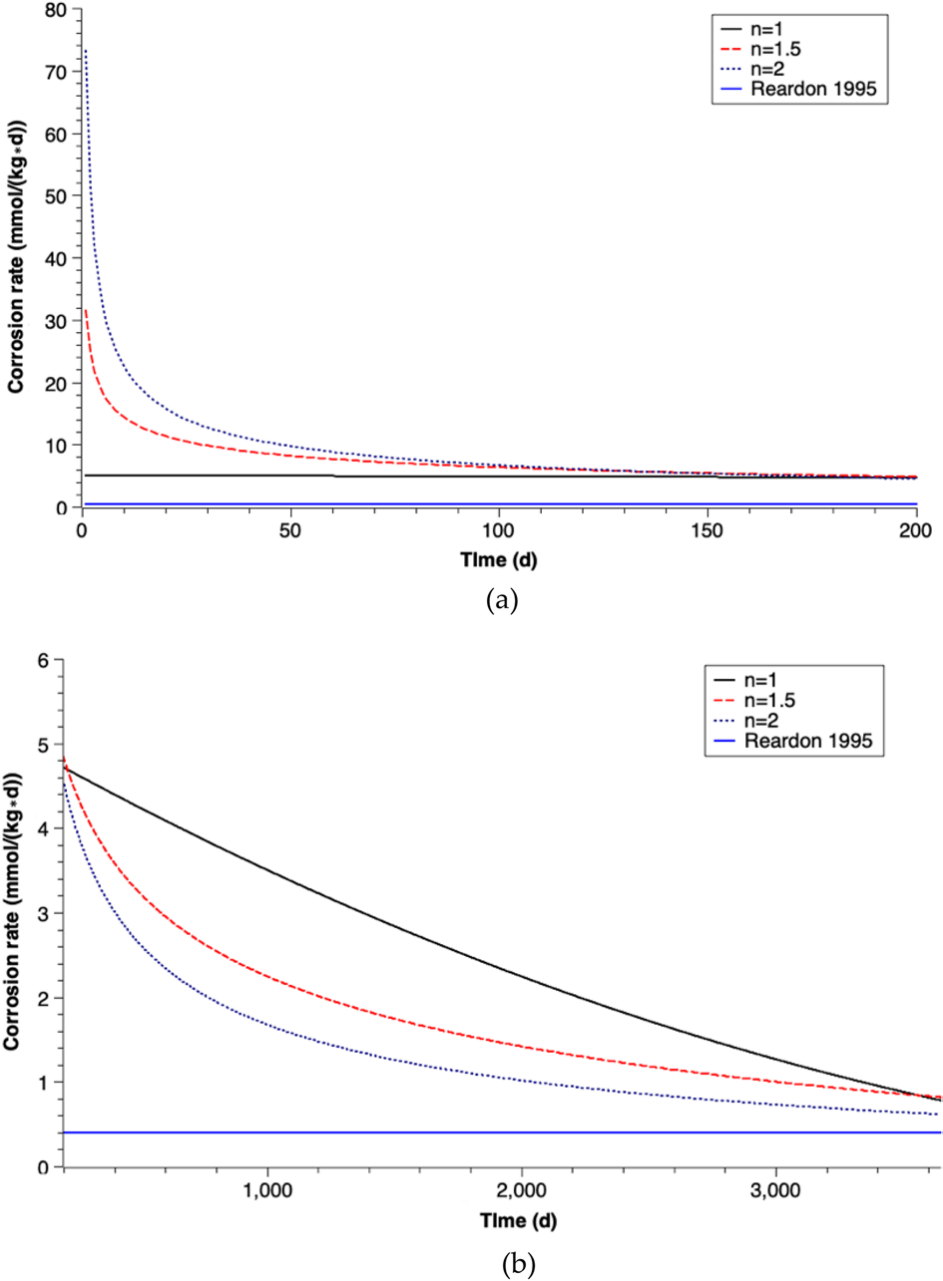
Comparison of corrosion rates with results from Reardon^44^ (n is the coefficient of passivation) assuming goethite as reaction product (**a**) 0–200 days, (**b**) 200–3650 days.

It is difficult to compare the corrosion rate results of this study with those of previous studies, since different units of corrosion rate were used (e.g. mmol/(kg day), mol/(m^2^ day), etc.) in former studies. The most frequently used corrosion rate value in Fe^0^-based PRB modeling studies^20,30,41,50^ is derived from the report of Reardon et al.^44^. Reardon measured the iron corrosion rates by monitoring the hydrogen pressure increase in sealed cells containing iron granules and water. The corrosion rate was given in mmol/(kg day). The units of corrosion rates in this study are converted to mmol/(kg day) and the results are shown in Fig. 8a,b.

Differences of more than an order of magnitude are shown between the calibrated corrosion rates in this study and the data reported by Reardon^44^ at the beginning of corrosion. The initial corrosion rates with different coefficients of passivation (n) in this study are 5.0 mmol/(kg day) (n = 1), 31.4 mmol/(kg day) (n = 1.5) and 73.2 mmol/(kg day) (n = 2) respectively. The corrosion rates strongly decrease over time and the average corrosion rates of a 10 years simulation are 2.60 mmol/(kg day) (n = 1), 2.07 mmol/(kg day) (n = 1.5), and 1.77 mmol/(kg day) (n = 2) respectively. These rates are higher than the rate of 0.4 mmol/(kg day) for deionized water published by Reardon^44^ but fall within the range of reported corrosion rates of 0.2–50 mmol/(kg day)^49^.

The relatively low corrosion rates reported by Reardon 1995 contradicts to the porosity reduction (57–32%) observed by Luo et al.^58^. In both cases, deionized water was in contact with granular iron. The possible reasons for this remarkable difference are (1) Reardon measured the corrosion rates with a batch experiment with no water flow (rate = 0). But in typical PRB systems, the underground water flows through the barrier under the natural hydraulic gradient, which increases the iron corrosion rate^70^. (2) Reardon monitored the hydrogen pressure increase in sealed cells. The increasing hydrogen partial pressure may inhibit the iron corrosion reaction. For a real site PRB, the generated hydrogen can escape immediately from the system. (3) The intrinsic reactivity of iron materials varies significantly which may cause an order magnitude difference in corrosion rate^71^.

Model approaches on the basis of data from Reardon^44^ underestimate the iron corrosion process as well as the influence of the iron corrosion products on the porosity of the PRB system during the long-term operation. With higher corrosion rates of iron, more iron will react in the water, and larger amount of iron corrosion products are generated. The increasing generated corrosion products can fill the pore in the barrier quickly and cause the early failure of the PRB technique.

Moreover, the reaction process is very complicated in the real PRB systems. The iron corrosion rate is easily influenced by many factors^20^ and can vary dramatically in different parts of the PRB. For example, dissolved oxygen (DO) is consumed once it enter the iron zone, and it can accelerate the iron corrosion process, which induces more corrosion products, and thus higher porosity reduction in the entrance zone of PRBs^27^.

## Considerations on reactive surface area change versus time

**Figure 9:**
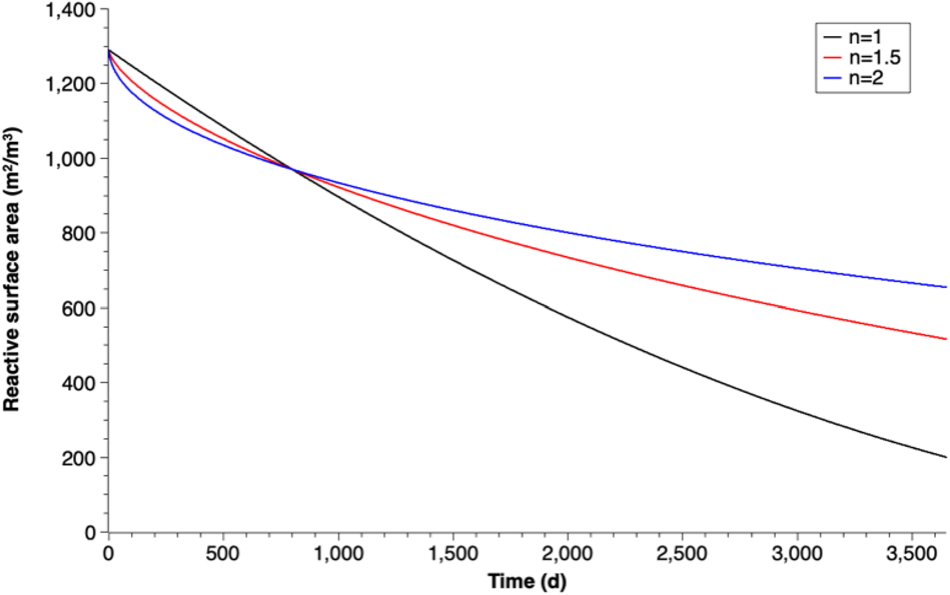
Reactive surface area of Fe^0^ particles versus time (n is the coefficient of passivation).

The reactive surface area of Fe^0^ in this study can be calculated by the depletion of the radius of the iron particles (Eq. 10) and the assumption of uniform corrosion. Plots of calculated reactive surface area of different coefficients of passivation (n) over time are shown in Fig. 9.

**Figure 10:**
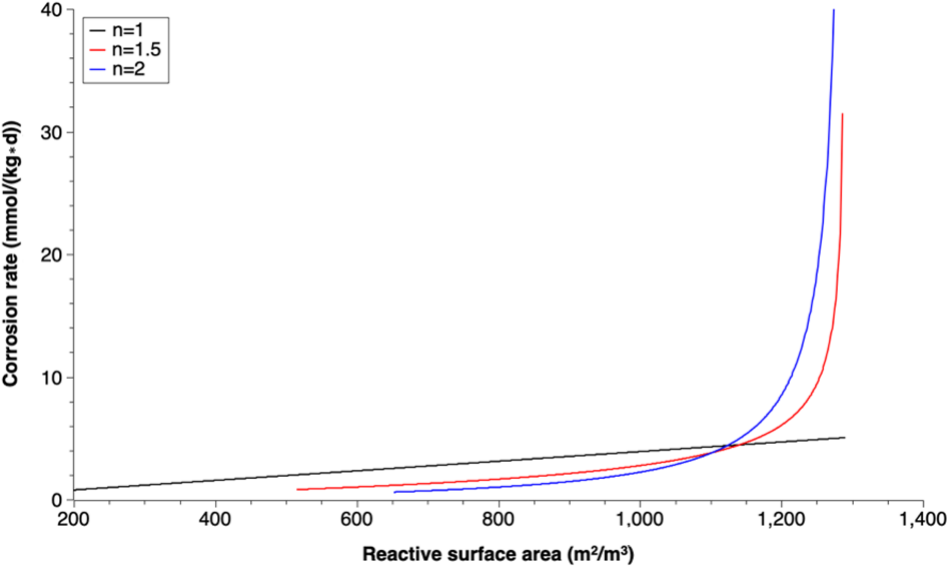
Corrosion rate versus reactive surface area with different coefficients of passivation (n).

For most of previous modeling studies on simulating the operation of Fe^0^-based PRBs, the reaction rate for iron corrosion by water was assumed to have a first-order dependency on the iron surface area^20,30,41,42,50,72–76^. A plot of calibrated corrosion rates versus the calculated reactive surface area values under different coefficients of passivation is shown in Fig. 10.

When no iron passivation considered, which means that the current density (i) or the corrosion rate (in mm/year) are constant, the corrosion rate in mmol/(kg day) has a complete first-order dependency on the iron surface area (Fig. 10). However, when the growth of corrosion products follows a parabolic law, the first-order dependence assumption is no longer applicable. The first order model underestimates the iron corrosion rate at large reactive surface area in the system. As shown in Fig. 10, the corrosion rate results for coefficient of passivation (n) is 1.5 or 2 are significantly larger than that of first-order dependency (n = 1) when the system has a large reactive surface area. This is valid especially during the beginning phase with strong increase of iron corrosion products, which causes the porosity reduction in the barrier. Thus, a more accurate expression of corrosion rate should be applied in modeling simulations in order to have a better estimation of PRB endurance.

## Comparison of porosity loss in different studies

Table 2 summarized the porosity loss simulations of previous studies with the simulations in this study with assumptions that the corrosion rate (in mm/year) is a constant and goethite is the only corrosion product.

**Table 2:** Porosity loss simulations in different studies.

Study	Solution	Simulated porosity loss after 1 year (%)
This study	Deionized water	12.3
Mayer et al.^41^	Underground water	0.7
Yabusaki et al.^42^	Underground water	2.56
Li et al.^20^	Underground water	0.65
Li et al.^77^	Underground water	1.2

The porosity loss after 1 year simulation in this study is over one order of magnitude larger than the simulation results from former studies. The simulated porosity loss from these studies contradict to the measured porosity loss reported by Luo et al.^58^. Possible reasons for the divergence are the different iron corrosion rates utilized in the model as discussed in “Comparison of corrosion rates in different studies”. In addition, this study simulated the condition that the granular iron is in contact with deionized water and only the iron corrosion process is considered. If the deionized water is replaced by underground water with multiple dissolved ions, the porosity loss, i.e. by precipitation of carbonates, can be even more significant.

## Effect of Fe^0^ mixing ratio

Figure 5 shows the simulated long-term porosity loss for systems with different Fe^0^ mixing ratios. Table 3 summarized the reported porosity values after the column experiments in Luo et al.’s study^58^ and the simulated porosity values after an equivalent reaction period.

**Table 3:** Porosity values for different Fe^0^ mixing ratios.

Fe^0^ mixing ratio (w/w, %)	Porosity (%) from Luo et al.^58^	Simulated porosity (%)
10	56	56
50	40	50
100	32	32

The results from column experiments in Luo et al.^58^ confirm that the system with lower Fe^0^ mixing ratio in the barrier can remain higher porosity after exposure to water. The higher porosity can be explained that a lower percentage of Fe^0^ generates less corrosion products, which reduce the likelihood of pore clogging in the system. Therefore, mixing Fe^0^ and less reactive materials (e.g. sand) is a solution for long-term porosity loss in Fe^0^-based PRBs^77^. However, a low Fe^0^ mixing ratio might reduce the ability of a PRB system to remove contaminants^58^. Thus an appropriate ratio between Fe^0^ and less reactive materials is important.

## Conclusion

A mathematical model is presented to simulate the long-term porosity loss of Fe^0^-based PRBs as induced by deionized water. It is assumed that only the volumetric expansive corrosion of iron contributes to the porosity loss of the system. Faraday’s law was applied to describe the correlation of the amount of corroded iron and the iron corrosion rates. Different coefficients of passivation were taken into account to describe different growth features of corrosion products. Measured porosity results from Luo et al.^58^ were used to calibrate the parameters in the model. Based on experimental findings from literature and the simulations here, the following major conclusions can be dawn.

- There are iron residues in the system (0.09 m^3^ Fe^0^/m^3^) when the porosity reduces to 0, which means the groundwater can no longer flow through the Fe^0^-based PRB before the Fe^0^ is completely consumed. Thus, it is not correct to assume that the iron in Fe^0^-based PRB is totally consumed and that the endurance of PRB can be estimated from the amount of iron and iron corrosion rate.
- The derived iron corrosion rates in presented model (2.60 mmol/(kg day), 2.07 mmol/(kg day) and 1.77 mmol/(kg day)) are significantly larger than the corrosion rate used in previous studies (0.4 mmol/(kg day)). Higher iron corrosion rate means more iron can dissolve in the water, which leads to more significant porosity loss caused by larger amount of generated iron corrosion products. Thus, the previous simulations with low iron corrosion rate may underestimate the porosity loss in PRB. Moreover, we propose, a uniform unit of iron corrosion rate (e.g. mm/year) for Fe^0^-based PRB systems in order to improve the comparability of the different studies.
- The assumption in previous modeling studies, which describes the iron corrosion rate (in mmol/(kg day)) as a first-order dependency on iron surface area, is accurate only when iron passivation is neglected. When iron passivation is considered, such an assumption underestimates the corrosion rates especially at the beginning phase of operation.
- The modelled porosity loss in this study (0.12/year with assumptions that the corrosion rate is a constant and goethite is the only corrosion product) is larger than the simulation results from previous studies (average 0.02/year). Our study demonstrates that iron corrosion products can cause large porosity loss in the filter. Iron passivation features and possible corrosion products are responsible for large differences between the simulation results. Therefore, iron corrosion processes need to be properly considered in order to accurately estimate the long-term operation of Fe^0^-based PRB systems.
